# Dynamic Changes in DNA Methylation Occur during the First Year of Life in Preterm Infants

**DOI:** 10.3389/fendo.2016.00158

**Published:** 2016-12-15

**Authors:** Chinthika Piyasena, Jessy Cartier, Nadine Provençal, Tobias Wiechmann, Batbayar Khulan, Raju Sunderesan, Gopi Menon, Jonathan R. Seckl, Rebecca M. Reynolds, Elisabeth B. Binder, Amanda J. Drake

**Affiliations:** ^1^British Heart Foundation Centre for Cardiovascular Science, The Queen’s Medical Research Institute, University of Edinburgh, Edinburgh, UK; ^2^Neonatal Unit, Simpson Centre for Reproductive Health, Royal Infirmary of Edinburgh, Edinburgh, UK; ^3^Department of Translational Research in Psychiatry, Max-Planck Institute of Psychiatry, Munich, Germany

**Keywords:** prematurity, DNA methylation, IGF2, FKBP5, glucocorticoids

## Abstract

**Background:**

Preterm birth associates with a substantially increased risk of later cardiovascular disease and neurodevelopmental disorders. Understanding underlying mechanisms will facilitate the development of screening and intervention strategies to reduce disease risk. Changes in DNA methylation have been proposed as one mechanism linking the early environment with later disease risk. We tested the hypothesis that preterm birth associates with altered DNA methylation in genes encoding insulin-like growth factor 2 (IGF2) and FK506-binding protein 5 (FKBP5), which appear particularly vulnerable to early life adversity.

**Methods:**

Fifty preterm infants were seen and assessed at birth, term equivalent age, 3 months and 1-year corrected ages; 40 term infants were seen at birth, 3 months and 1 year. Saliva was collected for DNA extraction at birth, term, and 1 year. Pyrosequencing of bisulfite-converted DNA was performed to measure DNA methylation at specific CpG sites within the *IGF2* and *FKBP5* loci.

**Results:**

Weight and head circumference was reduced in preterm infants at all time points. Preterm infants had a higher percentage body fat at term-corrected age, but this difference was not persistent. DNA methylation at the differentially methylated region (DMR) of *IGF2* (*IGF2DMR2*) and *FKBP5* was lower in preterm infants at birth- and term-corrected age compared to term infants at birth. *IGF2DMR2* and *FKBP5* methylation was related to birthweight SD score in preterm infants. Among preterm infants, social deprivation was an independent contributor toward reducing DNA methylation at *IGF2DMR2* at birth- and term-corrected age and maternal smoking was associated with reduced DNA methylation at *FKBP5* at birth. There were no persistent differences in DNA methylation at 1 year of age.

**Conclusion:**

Changes in DNA methylation were identified at key regions of *IGF2/H19* and *FKBP5* in preterm infants in early life. Potential contributing factors include maternal smoking and social deprivation. However, these changes did not persist at 1 year of age and further longitudinal studies are required to determine any associations between altered DNA methylation in the perinatal period of individuals born preterm and their long-term health.

## Introduction

Epidemiological evidence linking low birthweight with an increased risk of cardiovascular disease as well as developmental neuropsychiatric disorders (1) has led to the concept of “early life programming.” This proposes that exposure to adverse conditions during critical stages of early development results in a change in the offspring structural and functional phenotype (2). Preterm birth acts as a profound challenge in early life. There is now substantial evidence that prematurity associates with risk factors for cardiovascular disease in adulthood, including hypertension and insulin resistance (3–5). Furthermore, preterm birth is closely associated with neurodevelopmental disorders including cognitive impairment and autism spectrum disorder (6). This has important implications for public health, since worldwide, 15 million infants are born preterm every year, and survival rates have increased markedly over recent years (7).

Factors acting during intrauterine development, which may be important in mediating programing effects in infants born small at term, include undernutrition and glucocorticoid overexposure. These may be of particular importance in infants born preterm. In addition, preterm infants are vulnerable to these factors acting in early *postnatal* life (4). Following birth, many preterm infants develop a cumulative protein and energy deficit and exhibit early postnatal growth failure (8). Preterm infants are additionally exposed to repeated stressful and often painful procedures during a period of rapid brain maturation, and several studies have shown an impact of these procedures on neurodevelopment and hypothalamic–pituitary–adrenal (HPA) axis activity (9, 10).

Understanding the mechanisms by which prematurity associates with long-term effects on health would facilitate the development of effective screening and intervention strategies. Changes in DNA methylation have been proposed as one mechanism linking early life events and later disease risk (11), and genome-wide profiling has revealed DNA methylation differences between preterm and term infants in early life (12–14). Exposure to an adverse environment in early life has repeatedly been associated with altered DNA methylation at a gene of particular importance for fetal growth: the imprinted gene insulin-like growth factor 2 (IGF2). *IGF2* is a key growth factor, particularly in early development. *IGF2* expression is controlled by DNA methylation at a number of differentially methylated regions (DMRs) (15) and altered *IGF2* DNA methylation has been reported following exposure to altered maternal nutrition including severe famine (16, 17).

The early life environment can also impact on the normal functioning of the HPA axis, with implications for neurodevelopment. Exposure to an adverse environment pre- or postnatally has been associated with altered DNA methylation at a number of genes important in determining HPA axis function, including the glucocorticoid receptor (*GR*) and 11beta-hydroxysteroid dehydrogenase type 2 (*11β-HSD2*). Differences in DNA methylation at *GR* and *11β-HSD2* in placenta have been reported in association with infant behavioral development (18); however, we have previously reported that DNA methylation at *GR* and *11β-HSD2* is extremely low in individuals exposed to an adverse early life environment and is, therefore, unlikely to impact on gene expression (17). FK506-binding protein 5 (*FKBP5*) encodes a co-chaperone of GR and is induced following stress exposure through GR binding to specific genomic response elements; in turn, FKBP5 protein binds to the GR complex, reducing its affinity for cortisol and decreasing nuclear translocation (19). Thus, it is an important component of the stress response. A functional polymorphism in *FKBP5* intron 2 alters mRNA and protein induction following GR activation (20), such that the allele associated with stronger *FKBP5* mRNA induction associates with GR resistance and an increased risk of a number of psychiatric disorders following childhood trauma (20). Exposure to childhood trauma leads to allele-specific epigenetic changes, with a GR-binding-induced decrease in DNA methylation within a functional glucocorticoid response element in intron 7, specifically, in carriers of the risk allele (20). Further, DNA methylation of FKBP5 in placenta associates with infant arousal scores (21).

In this study, we tested the hypothesis that preterm birth, a profound stressor in early life, associates with altered DNA methylation at the candidate loci IGF2 and FKBP5, which may be particularly vulnerable to early life adversity, and examined whether any changes were persistent over the first year of life.

## Materials and Methods

### Cohort

Fifty preterm (<32 weeks gestation) and 40 term (37–42 weeks gestation) infants were recruited within the first week of life from the Simpson Center for Reproductive Health, Edinburgh, UK, with informed written parental consent. Ethical approval was obtained from the South East Scotland Research Ethics Committee (Reference 11/AL/0329). NHS management approval was obtained (Lothian R&D Project number 2011/R/NE/03). Perinatal samples were collected under the Edinburgh Reproductive Tissue BioBank (ERTBB) (West of Scotland Research Ethics Service Reference 09/S0704/3). All parents gave written informed consent and all studies were performed in accordance with the declaration of Helsinki. Infant samples were collected under the framework of the ERTBB following an amendment to ethical approval (Reference AM07/1). Demographic details were obtained during clinic visits and from hospital records. All of the preterm infants were admitted to the neonatal unit; of these, six infants died. In three of these infants, saliva for buccal cells was not collected after birth due to clinical instability and DNA was of poor quality in a fourth infant. Five of the term infants were also admitted to the neonatal unit for a short period (respiratory distress syndrome, weight loss, and hemolysis from Rhesus isoimmunization) but none required follow-up. Preterm infants were seen at birth, at term-corrected age, and at 3 months and 1-year corrected ages; term infants were seen at birth, 3 months, and 1 year. All visits occurred in the afternoon, supervised by one researcher (Chinthika Piyasena).

### Growth and Body Composition

Weight, length, and occipitofrontal head circumference (OFC) was measured by one trained researcher. Percentage body fat mass was measured by air displacement plethysmography in preterm infants at term-corrected age and 3 months corrected age, and in term infants at birth and 3 months using the PEAPOD Body Composition System (COSMED, Chicago, IL, USA). Skin fold thickness (subscapular and triceps) was measured at 1 year by the same trained researcher. Term infants were measured at a median of 2 days (range 0–8) after birth, at 3 months (13.3 weeks; range 10.4–16.9), and 1 year (52.3 weeks; range 48.1–57.4). Preterm infants were measured at term-corrected age at a median of 40 (range 35 + 0 to 44 + 1)-corrected weeks, at 3 months corrected (13.4 weeks; range 10.3–18.3) and 1 year corrected (53.9 weeks; range 52.1–68.7).

### Analysis of DNA Methylation

Saliva was collected using the Oragene DNA (OG-250) kits and saliva sponges CS-1 and extracted using prepIT-2LP (DNA Genotek, Ottawa, ON, Canada). DNA was quantified using the Qubit 2.0 Fluorometer (Life Technologies, Paisley, UK). Five hundred nanograms of DNA were bisulfite converted using the EZ DNA Methylation Gold Kit (Zymo Research Corporation, CA, USA). Pyrosequencing was performed to analyze DNA methylation for DMRs controlling *IGF2* expression: *IGF2* DMR2 (*IGF2DMR2, n* = 9 CpGs) and the H19 imprinting control region (*H19ICR, n* = 8 CpGs) as previously described (22). Primers were purchased from Invitrogen (Life Technologies, Paisley, UK). DNA was amplified using the AmpliTaq Gold 360 kit (Applied Biosystems, Warrington, UK) and pyrosequencing performed using PyroMark Q24Gold reagents on a PyroMark Q24 Pyrosequencer (Qiagen, Crawley, UK). Data were analyzed using PyroMark Q24 1.0.10. Percentage DNA methylation is expressed as the average across all CpGs in each of the two loci in *IGF2*.

Methylation analysis of three CpGs in *FKBP5* intron 7, two of which are located in consensus GRE motif (CpG 2 and 3), was performed in triplicates using a protocol adapted from Klengel et al. (20). One hundred twenty nanograms genomic DNA was bisulfite converted using the EZ DNA Methylation Kit (Zymo Research Corporation, CA, USA). Bisulfite converted DNA was amplified in a 50 µl reaction mix (4–10 µl DNA; each bisulfite specific Primer with a final concentration of 0.2 µM, FKBP5int7_P1_F: GTTGTTTTTGGAATTTAAGGTAATTG, and FKBP5int7_P1_R_biot: biotin-TCTCTTACCTCCAACACTACTACTAAAA) using the Kapa HIFI Uracil + Hot start Ready Mix (Kapa Biosystems Inc., Wilmington, DE, USA). Cycling conditions of the touchdown PCR were 98°C for 5 min, 2× (98°C – 40 s, 62°C – 30 s, 72°C – 60 s), 5× (98°C – 40 s, 60°C – 30 s, 72°C – 60 s), 8× (98°C – 40 s, 58°C – 30 s, 72°C – 60 s), 34× (98°C – 40 s, 56°C – 30 s, 72°C – 60 s), 72°C for 1 min and cooling to 4°C. Pyrosequencing of *FKBP5* CpGs was performed on a PyroMark Q96 ID system (QIAGEN GmbH, Hilden) using PyroMark Q96Gold reagents with the following sequencing primer: FKBP5int7_P1_S2: 5′-GTTGATATATAGGAATAAAATAAGA-3′ for CpG1 and CpG2 and FKBP5int7_P1_S3: 5′-TGGAGTTATAGTGTAGGTTTT-3′ for CpG3. PyroMark Q96 ID Software 2.5 (QIAGEN GmbH, Hilden) was used to calculate percentage methylation.

**Figure 1:**
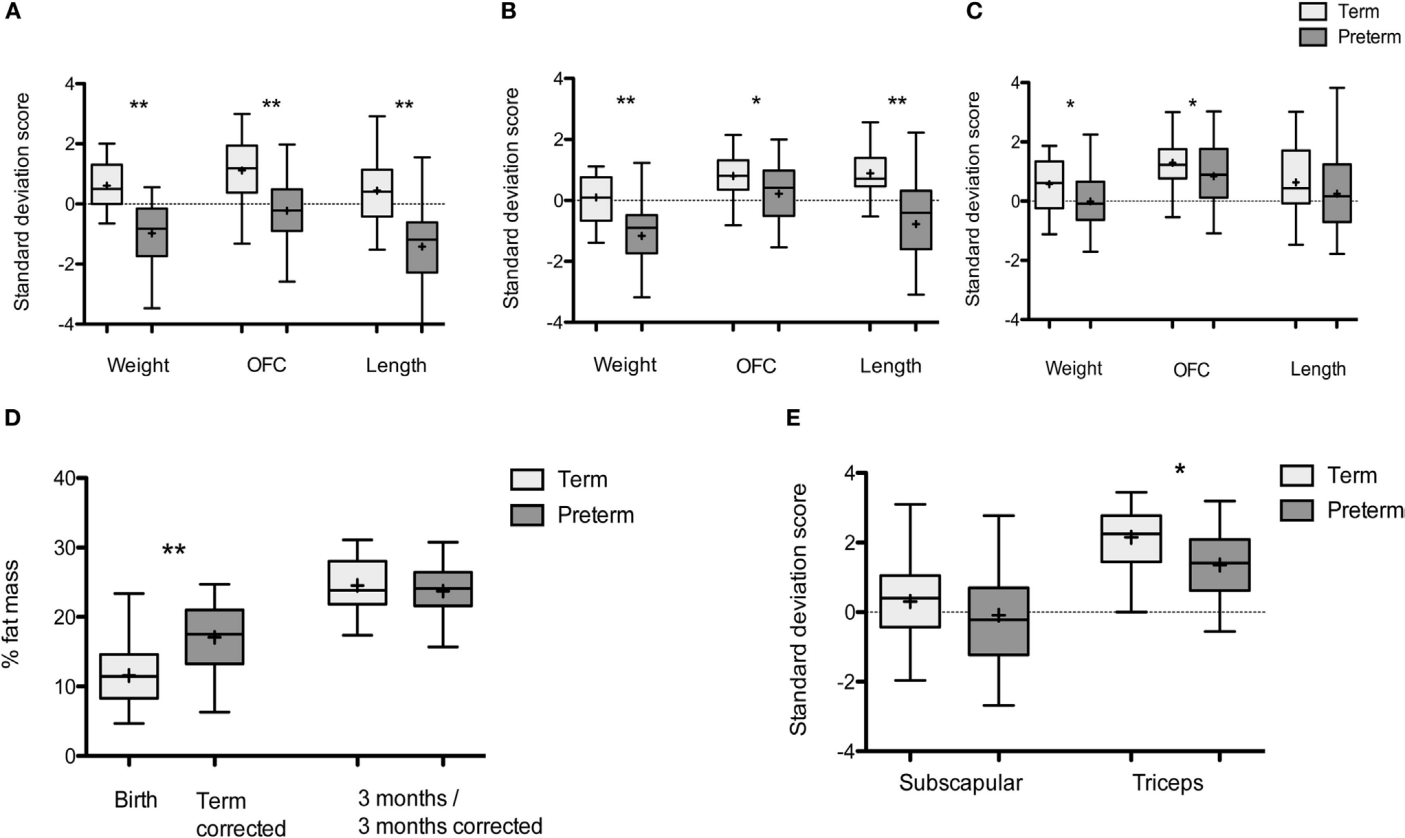
**Growth of term and preterm infants over the first year: box and whisker plots: Tukey with line at median (+ indicates mean)**. **(A)** Weight, OFC, and length at birth- and term-corrected age. Term infants: *n* = 40, preterm infants: *n* = 43. **(B)** Weight, OFC, and length at 3 months/3 months-corrected age. Term infants *n* = 35, preterm infants *n* = 35. **(C)** Weight, OFC, and length at 1 year/1-year corrected. Term infants *n* = 35, preterm infants *n* = 42. **(D)** % fat mass in preterm and term infants at birth- and term-corrected age and 3 months of age. Term infants: *n* = 32 at birth and 30 at 3 months, preterm infants: *n* = 21 at term-corrected age and 32 at 3 months corrected. **(E)** Skinfold thickness in term and preterm infants at 1 year/1-year corrected. Term infants *n* = 32, preterm infants, *n* = 31 (*n* denotes the number of measurements obtained). *p < 0.05, **p < 0.001.

**Figure 2:**
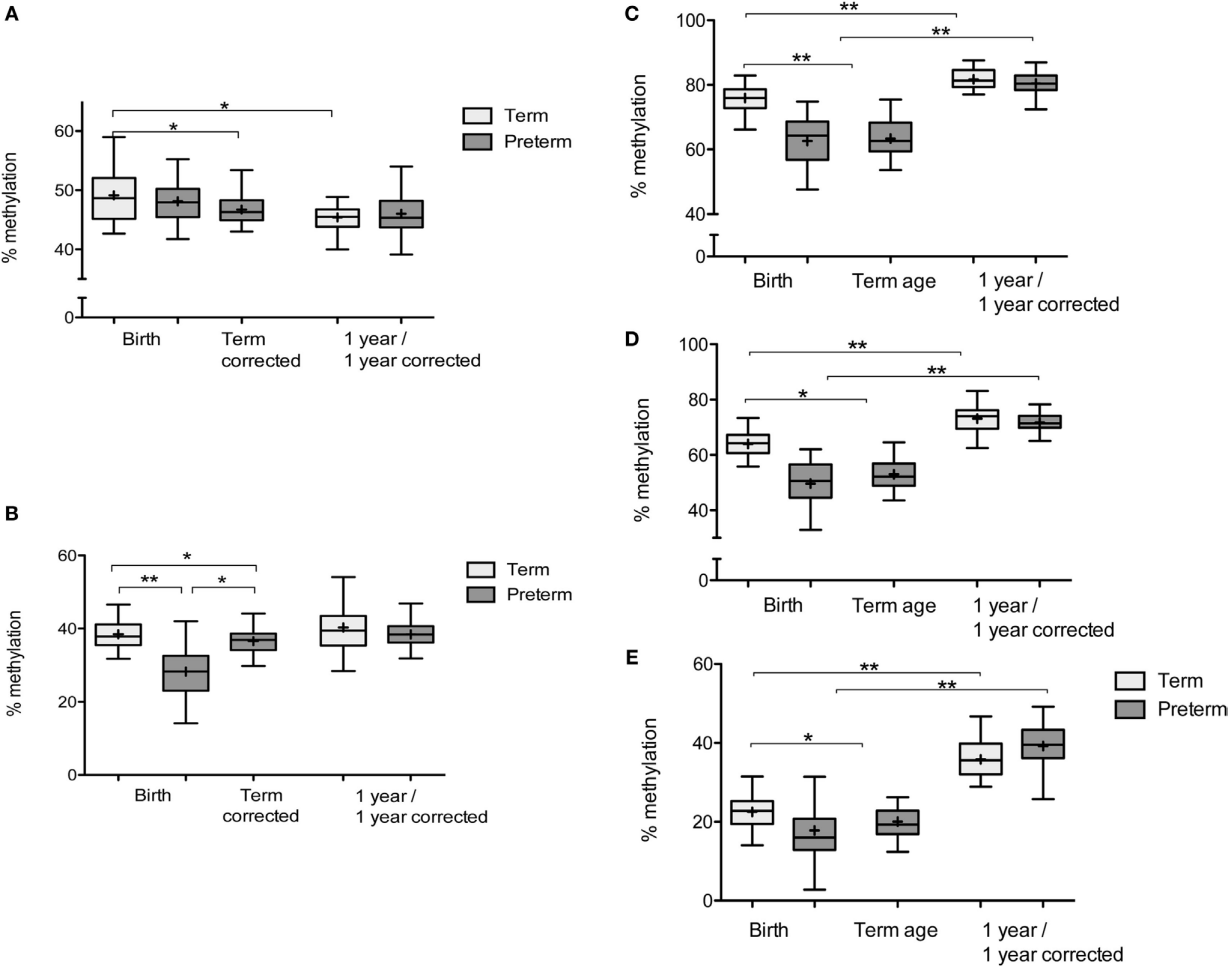
**Percentage DNA methylation at *IGF2/H19* and *FKBP5*: box and whisker plots: Tukey with line at median (+indicates mean)**. **(A)** % DNA methylation at *H19ICR*: term infants: *n* = 28 at birth and 31 at 1 year. Preterm infants: *n* = 23 at birth, 31 at term corrected, and 32 at 1-year corrected. **(B)** % DNA methylation at *IGF2DMR2*: term infants: *n* = 25 at birth and 23 at 1 year. Preterm infants: *n* = 22 at birth, 32 at term corrected, and 28 at 1-year corrected. **(C)** DNA methylation at FKBP5 CpG1: term infants: *n* = 32 at birth and 31 at 1 year. Preterm infants: *n* = 27 at birth, 37 at term-corrected age, and 32 at 1-year corrected. **(D)** DNA methylation at FKBP5 CpG2: term infants: *n* = 28 at birth and 30 at 1 year. Preterm infants: *n* = 25 at birth, 33 at term-corrected age, and 32 at 1-year corrected. **(E)** DNA methylation at FKBP5 CpG3: term infants: *n* = 30 at birth and 30 at 1 year. Preterm infants: *n* = 27 at birth, 36 at term-corrected age, and 36 at 1-year corrected (*n* denotes the number of measurements obtained). *p < 0.05, **p < 0.001.

The legends for Figures 1 and 2 state the number of values/measurements analyzed, excluding samples, which were discarded because of insufficient DNA and/or poor pyrosequencing quality. Additionally, we were unable to collect saliva from several babies for various reasons, although these babies still underwent anthropometry.

### FKBP5 SNP Genotyping

*FKBP5* rs1360780 genotyping was performed on a Roche LightCycler 480 System using a TaqMan SNP Genotyping Assay (Applied Biosystems). Thermal cycling conditions were 95°C for 10 min, 45× (95°C – 15 s, 60°C – 1 min, 50°C – 30 s). Genotypes were called using endpoint genotyping LightCycler 480 Software version 1.5. Genotypes were in Hardy–Weinberg equilibrium (*p* = 0.17). Participants were divided into protective–genotype (CC) and risk-allele carriers (CT or TT).

### Covariates

Covariates that could confound the association or be in the causal pathway were added into the model in a hierarchical manner: maternal smoking, male gender, and social deprivation for *FKBP5* and *IGF2/H19*. Additionally, breast milk at 3 months for *IGF2/H19* at 1 year and risk-allele carriage for *FKBP5* at all time points. Social deprivation was coded as deprivation category (DEPCAT) scores based on the mother’s postcode at booking and obtained from the “Carstairs scores for Scottish postcode sectors from the 2001 Census.” In this system, scoring is based on the material affluence/deprivation of the area in which a person lives. Postcode sectors are allocated a DEPCAT score, derived from four sets of information: overcrowding, male unemployment, car ownership, and the proportion of people in households in social class 4 or 5 and scores range from 1 to 7 where 7 indicates the worst social deprivation. Maternal smoking was categorized as current smoker, never smoked, former (stopped pre-pregnancy), or former (stopped during pregnancy). Breast milk at 3 months indicates whether or not the infant was receiving any breast milk at 3 months corrected age.

### Statistics

Weight, length, OFC, skin fold thickness, and weight gain were adjusted for age and gender by converting to SD scores (SDS) (*z*-scores) using LMSgrowth, a Microsoft Excel Add-in to access growth references that define the UK-WHO growth charts (23). Demographic, clinical characteristics, and risk-allele carriage between preterm and term infants were compared using independent samples *t*-testing and chi-square analyses, as appropriate. Multivariate linear regression was used to assess variation in body composition and to test the hypothesis that preterm birth is associated with altered DNA methylation. Outcome variables were percentage DNA methylation at birth, term-corrected age, and 1 year. Unstandardized regression (β) coefficients from these models indicate the change in percentage methylation associated with prematurity and a one-unit change in the other predictors. Paired samples *t*-testing was used where appropriate. One-way ANOVA with Dunnett’s *post hoc t* testing was used to test the effect of maternal smoking on DNA methylation at *FKBP5*. Statistical significance for all analyses was set at *p* < 0.05 (two-tailed).

## Results

### Preterm and Term Infant Demographics

Characteristics of the cohort are shown in Table 1. Birth weight SDS was lower in preterm infants *p* < 0.001, and there were more males in the preterm group, *p* = 0.006. There was no significant difference between the frequency of *FKBP5* risk-allele carriage between the groups (*p* = 0.43). Maternal age, body mass index (BMI), DEPCAT scores, folic acid in first trimester, and maternal smoking were different between the two groups.

**Table 1:** **Characteristics of the study participants**.

	Term	Preterm	*p*-Value
**Infant characteristics**			
Gestation at birth, weeks	40.2 ± 1.1	28.5 ± 2.1	<0.001
Birth weight, g	3,649 ± 517	1,136 ± 350	<0.001
Birth weight SDS	0.44 ± 1.0	−0.44 ± 0.9	<0.001
Male, *n* (%)	15 (38)	35 (70)	0.006
Death, *n* (%)	0	6 (12)	
Bronchopulmonary dysplasia, *n* (%)	0	14 (31)	
Laser for retinopathy of prematurity, *n* (%)	0	5 (11)	
Necrotizing enterocolitis, *n* (%)	0	6 (13.3)	
Intraventricular hemorrhage, *n* (%)	0	3 (6)	
Periventricular leucomalacia, *n* (%)	0	2 (4.2)	
Late onset sepsis, *n* (%)	0	17 (37)	
TPN duration, days	0	17.8 ± 21.8	
Any breast milk at 3 months, *n* (%)	28 (75.7)	9 (22.5)	<0.001
*FKBP5* risk allele carriage, *n* (%)	21 (52.5)	28 (60.9)	0.43
**Maternal characteristics**			
Age, years	35.2 ± 4.6	31.3 ± 6.2	0.001
Body mass index at booking, kg/m^2^	24.3 ± 3.1	27.2 ± 6.9	0.016
High DEPCAT score, *n* (%)	11 (27.5)	27 (61.4)	0.002
Caucasian ethnicity, *n* (%)	40 (100)	41 (93.8)	0.09
Smoking, *n* (%)			
*Current*	0	10 (22.7)	
*Former – stopped during pregnancy*	2 (5)	4 (9.1)	0.5
*Former – stopped pre-pregnancy*	12 (30)	11 (25)	0.61
*Never*	26 (65)	18 (40.9)	0.03
Primiparity, *n* (%)	21 (52.5)	29 (65.9)	0.69
Folic acid during first trimester, *n* (%)	40 (100)	38 (86.3)	<0.001
Assisted reproduction, *n* (%)	0	6 (13.6)	
Multiple pregnancy, *n* (%)	0	9 (20.4)	
Hypertension or preeclampsia, *n* (%)	0	10 (22.7)	
Diabetes during pregnancy, *n* (%)	0	3 (6.8)	
Antenatal steroids, *n* (%)			
*None*	40 (100)	3 (6.8)	<0.001
*Incomplete course*	–	11 (25)	
*Complete course*	–	30 (68.2)	
Antenatal magnesium sulfate, *n* (%)	0	10 (22.7)	
Cesarean section, *n* (%)	28 (70)	23 (52.2)	0.1
Labor, *n* (%)	18 (37.5)	27 (61.4)	0.13
Age of partner	35.3 ± 5.2	33.1 ± 6.9	0.1

*Plus–minus values are means ± SD. *n* = 40 full term and 50 preterm infants. *n* = 44 for mothers of preterm infants. Bronchopulmonary dysplasia (BPD) is defined as need for respiratory support and/or supplemental oxygen at 36 weeks’ postmenstrual age to maintain oxygen saturations of 90% or more. A diagnosis of necrotizing enterocolitis was applied when cases achieved Bell stage 2 or greater. Intraventricular hemorrhage is defined as only grades III or IV events. The definition of late onset sepsis is taken from the Vermont Oxford Network Manual of Operations, release 16.3. Five preterm infants died before 36 weeks’ postmenstrual age (mean 24.9 weeks’ gestational age at birth, mean 692 g birthweight). A further preterm infant died at term-corrected age from severe BPD (26-weeks’ gestation at birth weighing 1,000 g). In three of these infants, saliva for buccal cells was not collected after birth due to clinical instability and DNA was of poor quality in a fourth infant. Three term infants and six preterm infants were not seen at 3 months. High DEPCAT was regarded as scores 4–7*.

*SDS, SD score; DEPCAT, deprivation category*.

### Growth and Body Composition

Weight and OFC was reduced in preterm infants at all time points compared to term infants (Figures 1A–C). Preterm infants were shorter than term infants at term-corrected age and 3 months corrected age, but not at 1-year corrected (Figures 1A–C). Preterm infants had a higher percentage body fat at term-corrected age when compared with term infants at birth (Figure 1D) (mean difference 5.5%, 95% CI [8.0, 3.0], *p* < 0.001) and this remained significant following adjustment for gender (β = 5.7, 95% CI [3.1, 8.3], *p* < 0.001). The difference in percentage body fat did not persist at 3 months/3 months corrected, including when adjusted for gender and breast milk intake (β = −0.3, 95% CI [−2.8, 2.2], *p* = 0.82) (Figure 1D). At 1-year corrected, preterm infants had lower triceps skin fold thickness SDS than term infants, adjusted for breast milk exposure at 3 months (β = −0.4, 95% CI [−1.3, −0.3], *p* = 0.008), but there were no differences in subscapular skin fold thickness (Figure 1E).

### Prematurity and DNA Methylation

Mean DNA methylation at *IGF2DMR2* was lower in preterm infants at birth compared to term infants at birth and this remained significant in adjusted analyses (β = −11.2, 95% CI [−15.2, −7.3], *p* < 0.001) (Figure 2B). There was no difference in mean DNA methylation at the *H19ICR* between preterm and term infants at birth after adjusted analysis (β = −1.3, 95% CI [−3.8, 1.2], *p* = 0.3) (Figure 2A). In preterm infants at term-corrected age, DNA methylation was reduced at *IGF2DMR2* and *H19ICR* compared to infants born at term, and this remained significant in adjusted analyses (β = −2.8, 95% CI [−5.0, −0.6], *p* = 0.01 and β = −2.3, 95% CI [−4.6, −0.1], *p* = 0.049, respectively) (Figures 2A,B). However, the significance was attenuated when social deprivation was added into the model for *IGF2DMR2* (β = −2.1, 95% CI [−4.3, 0.05], *p* = 0.055). At 1 year of age, there was no difference in mean DNA methylation in adjusted analyses at either *IGF2DMR2* (β = −0.3, 95% CI [−4.0, 3.3], *p* = 0.86) or *H19ICR* (β = 0.8, 95% CI [−1.5, 3.0], *p* = 0.49) (Figures 2A,B).

The presence of the *FKBP5* risk allele was associated with higher DNA methylation at *FKBP5* CpG3 at birth (β = 4.0, 95% CI [0.3, 7.6], *p* = 0.03) and term-corrected age (β = 2.5, 95% CI [0.4, 4.5], *p* = 0.02) across all infants. DNA methylation at CpG1, CpG2, and CpG3 was lower in preterm infants at birth and at term-corrected age when compared to term infants at birth, including after adjusted analysis (Table 2). There was an increase in DNA methylation at all three CpGs in both groups during the first year (Figures 2C–E) and, by 1 year of age, there were no persistent differences in mean methylation at any CpG between preterm and term infants (Table 2). The presence/absence of the *FKBP5* risk allele as a covariate did not alter the findings at 1 year and there was no moderation of the relationship between prematurity and DNA methylation by the presence of the risk allele at any time.

**Table 2:** **DNA methylation at *FKBP5* in preterm and term infants**.

A	CpG1		CpG2		CpG3	
	β	*p*	β	*p*	β	*p*
Preterm infants at birth vs. term infants at birth	−12.1 [−15.6, −8.8]	<0.001	−12.9 [−16.9, −9.0]	<0.001	−5.2 [−9.3, −1.2]	0.01
Preterm infants at term-corrected age vs. term infants at birth	−11.7 [−14.6, −8.7]	<0.001	−7 [−11.5, −2.5]	0.003	−3 [−5.3, −0.7]	0.01
Preterm infants at 1-year corrected vs. term infants at 1 year	−1.9 [−4.0, 0.2]	0.07	−2 [−4.4, 0.4]	0.1	3.1 [0.8, 6.0]	0.05

*β values represent unstandardized regression coefficients adjusting for male gender, maternal smoking, social deprivation score, and risk-allele carriage. Values in square brackets represent 95% confidence intervals*.

### Body Composition and DNA Methylation

*IGF2DMR2* methylation was highly significantly related to birthweight SDS in preterm (*R* = 0.7, *p* < 0.001), but not term infants. DNA methylation across *FKBP5* was also positively associated with birthweight SDS (β = 0.3, 95% CI [0.3, 5.4], *p* = 0.03) when adjusted for prematurity, but only in the preterm infants when analyzed separately (*R* = 0.4, *p* = 0.04). There were no other significant relationships between DNA methylation at *IGF2DMR2, H19ICR*, or *FKBP5* and weight or measures of body composition in either preterm or term infants at any other time point (Table 3).

**Table 3:** **Correlations between percentage DNA methylation at *IGF2DMR2, H19ICR*, and *FKBP5* with weight SDS and percentage body fat**.

	*IGF2DMR*		*H19ICR*		*FKBP5* CpG1		*FKBP5* CpG2		*FKBP5* CpG3		Intron average	
	*R*	*p*	*R*	*p*	*R*	*p*	*R*	*p*	*R*	*p*	*R*	*p*
**% DNA methylation vs. weight SDS**												
Term infants at birth vs. birthweight SDS	0	0.98	−0.2	0.44	0	0.83	0.2	0.43	−0.2	0.22	0.2	0.37
Preterm infants at birth vs. birthweight SDS	0.7	<0.001	0.2	0.41	0.6	<0.001	0.5	0.01	0.4	0.04	0.4	0.04
Preterm infants at term age vs. weight SDS	0	0.96	0.2	0.37	0.2	0.34	−0.3	0.16	−0.1	0.53	0.1	0.73
Term infants at 1 year vs. weight SDS	−0.1	0.72	0.2	0.28	0	0.89	0.4	0.05	0.4	0.06	0.1	0.79
Preterm infants at 1 year corrected vs. weight SDS	−0.06	0.75	0.27	0.14	−0.2	0.29	−0.1	0.51	−0.2	0.22	0	0.86
**% DNA methylation vs. % body fat**												
Term infants at birth vs. % fat	0	0.94	−0.2	0.29	0	0.9	0	0.98	−0.2	0.4	0.2	0.27
Preterm infants at term age vs. % fat	−0.2	0.51	−0.2	0.62	0.3	0.17	0.2	0.47	0.2	0.48	−0.1	0.81

### Social Deprivation, Maternal Smoking, and DNA Methylation

Social deprivation was an independent contributor toward reducing DNA methylation at *IGF2DMR2* in preterm infants at birth (β = −1.6, 95% CI [−2.8, −0.3], *p* = 0.02) and at term-corrected age (β = −0.9, 95% CI [−1.5, −0.2], *p* = 0.02) although this did not persist at 1-year corrected age (*p* = 0.07). Social deprivation was also independently associated with a reduction in DNA methylation at *FKBP5* CpG2 in preterm infants at term-corrected age (β = −1.5, 95% CI [−2.9, −0.02], *p* = 0.047). Finally, maternal smoking was independently associated with a marked reduction in DNA methylation in preterm infants at *FKBP5* CpG1 (β = −2.6, 95% CI [−4.4, −0.7], *p* = 0.008) and CpG2 (β = −2.1, 95% CI [−4.1, −0.03], *p* = 0.047) at birth, compared to term infants. Additionally, maternal smoking was associated with a reduction in DNA methylation at birth in both groups at these loci (Table 4).

**Table 4:** **DNA methylation at FKBP5 according to maternal smoking status**.

Smoking status	CpG1			CpG2			CpG3		
	Mean	SEM	*p*-Value	Mean	SEM	*p*-Value	Mean	SEM	*p*-Value
**Birth (term and preterm infants)**									
Never	73.6	1.1		59.9	1.2		20.2	1.0	
Former stopped pre-pregnancy	66.1	2.4	0.007	56.4	2.7	0.49	21.4	2.3	0.91
Former stopped during pregnancy	68.8	3.0	0.47	57.0	5.3	0.90	20.8	3.4	1.00
Current	57.1	4.7	0.001	42.5	3.9	0.002	16.9	2.3	0.71
**Term age (preterm infants)**									
Never	62.9	1.4		53.1	1.6		20.4	1.0	
Former stopped pre-pregnancy	65.9	1.7	0.51	54.1	1.6	0.96	20.6	1.7	1.00
Former stopped during pregnancy	62.2	5.4	1.00	55.1	1.4	0.95	19.2	1.9	0.97
Current	61.6	2.0	0.95	50.6	2.1	0.71	18.1	1.0	0.66
**1 year (term and preterm infants)**									
Never	80.6	0.5		72.6	0.8		37.8	0.8	
Former stopped pre-pregnancy	81.4	1.1	0.82	72.1	0.9	0.97	37.2	1.5	0.97
Former stopped during pregnancy	83.6	1.4	0.23	72.5	1.6	1.00	36.4	2.6	0.91
Current	80.5	1.9	1.00	72.5	1.6	1.00	40.0	3.1	0.79

*Smoking status is recorded as never (regarded as the control group for one-way ANOVA and Dunnett’s *post hoc t* testing), former smokers who stopped before pregnancy, former smokers who stopped during pregnancy, and current (smokers who continued throughout pregnancy)*.

## Discussion

Preterm infants demonstrated a growth trajectory comparable to that reported in previous studies, such that they were lighter than term infants during the first year of life (24, 25). Although preterm infants had increased percentage fat mass at term-corrected age, there was no persisting difference at 3 months. Previous studies have reported increased and/or altered fat distribution in preterm infants at term-corrected age (26–28), with some reporting that these differences had resolved by 3 months (28). Despite this apparent resolution of excess adiposity in early life, preterm infants do show abnormalities of body composition in young adulthood, with higher adiposity, ectopic lipid deposition, and increased intra-abdominal fat (29).

DNA methylation at *IGF2DMR2* and *FKBP5* intron 7 was markedly lower in preterm infants at birth in comparison to term-born infants, and this was still the case at term-corrected age. Although we did not test the functional consequences of these alterations in DNA methylation, reduced *IGF2DMR2* DNA methylation would be predicted to reduce *IGF2* expression with potential implications for early growth. Reduced methylation of *FKBP5* intron 7 CpGs is associated with higher induction of *FKBP5* by *GR* activation, leading to increased *GR* resistance (20); however, whether this might play a role in the HPA axis dysregulation seen in preterm infants (9) is unclear. Social deprivation was an independent predictor of reduced methylation at *IGF2DMR2* at birth and term-corrected age. Inter-related factors such as smoking that accompany socioeconomic deprivation can impact on DNA methylation in adults (30), including at *FKBP5* (31). Recent studies in children and adults exposed to cigarette smoke *in utero* have reported alterations in global and site-specific DNA methylation (32–35), and our results showing that maternal smoking was independently associated with a marked reduction in DNA methylation at *FKBP5* at birth suggest that this extends to effects on *FKBP5*.

There were no persistent differences in DNA methylation at 1 year of age at the DMRs of *IGF2* and at *FKBP5*. Our data support an epigenome-wide association study (EWAS), which demonstrated that although there were many differences in DNA methylation between preterm and term babies at birth, these had largely resolved by 18 years of age. However, DNA methylation differences did persist at a subset of CpGs (12). This EWAS did not identify changes at IGF2 or FKBP5, which may reflect that it was small (*n* = 12 per group) and studied DNA methylation changes in blood spots, rather than buccal DNA. Our findings are also in agreement with longitudinal EWASs, which show that DNA methylation undergoes developmental changes during childhood (36). Notably, for *FKBP5*, DNA methylation increased between birth and 1 year in both term and preterm infants, and DNA methylation at CpG3 was still significantly lower at 1 year of age in comparison to levels described in adulthood (20), suggesting that there are ongoing changes in DNA methylation at this locus through childhood. Since studies clearly show that DNA methylation changes through infancy and childhood, it may be that the lower levels of DNA methylation in preterm infants at birth is a normal finding for infants at this gestation. For obvious reasons, we are unable to compare DNA methylation in saliva in infants of comparable gestation, who remained *in utero*. There were some persistent differences between term infants and preterm infants at term-corrected age, and we suggest that this may reflect differences between the intrauterine maturation in term-born infants and factors acting during the extra-uterine period to which preterm infants are exposed.

Alternative/additional explanations for the differences in DNA methylation between preterm and term infants in very early life could include the altered nutritional state of infants born preterm (37, 38); and/or glucocorticoid overexposure, since preterm infants experience both *in utero* exposure to synthetic glucocorticoids and significant early postnatal “stress.” *In vivo* and *in vitro* studies have shown that glucocorticoid exposure leads to stable DNA demethylation at these specific sites within the *FKBP5* locus (20, 39), and several studies have now shown that exposure to trauma during childhood associates with allele-specific demethylation at *FKBP5* in adulthood (20, 40). Although childhood abuse impacts on DNA methylation in carriers of the risk allele (20), we found no additional effects of the presence or absence of the risk allele. This is in agreement with a lack of effect of the risk allele on DNA methylation patterns following severe parental trauma (40), supporting the concept that these effects are specific to the timing of exposure (20), and it may be that the stresses around preterm birth occur outside this “vulnerable” period. Finally, it is also possible that differences in cell subtype populations in the saliva from preterm and term infants may impact on DNA methylation (41).

In summary, we found changes in DNA methylation at key regions of *IGF2/H19* and *FKBP5* in this cohort of preterm infants who did not achieve the reference for either growth or body composition by the time they reached 1 year of age. Further, we identified a number of contributing factors including maternal smoking and social deprivation. We found no persisting differences at 1 year of age. Whether further differences will emerge over longer follow-up is unknown; alterations in DNA methylation at key DMRs controlling the expression of *IGF2* have been reported in adults exposed to altered nutrition or prematurity (16, 17, 42); however, these were not longitudinal studies, so that any differences may have arisen postnatally. Additionally, these studies may have been complicated by the phenomenon of reverse causation, where the development of disease leads to changes in DNA methylation rather than *vice versa* (41). Further longitudinal studies are required to understand any potential long-term effects of early differences in DNA methylation in the perinatal period on the health of individuals born preterm.

## Ethics Statement

Infants were recruited within the first week of life from the Simpson Centre for Reproductive Health, Edinburgh, UK, with informed written parental consent. Ethical approval was obtained from the South East Scotland Research Ethics Committee (Reference 11/AL/0329). NHS management approval was obtained (Lothian R&D Project number 2011/R/NE/03). Perinatal samples were collected under the Edinburgh Reproductive Tissue BioBank (ERTBB) (West of Scotland Research Ethics Service Reference 09/S0704/3). All parents gave written informed consent and all studies were performed in accordance with the declaration of Helsinki. Infant samples were collected under the framework of the ERTBB following an amendment to ethical approval (Reference AM07/1).

## Author Contributions

CP and AD conceived the study. CP, GM, JS, and AD designed the study. CP, JC, BK, RS, NP, TW, RR, EB, and AD performed the study and analyses. CP and AD wrote the initial manuscript draft and all authors revised it critically for intellectual content. All authors gave final approval of the version to be published.

## Conflict of Interest Statement

The authors declare that the research was conducted in the absence of any commercial or financial relationships that could be construed as a potential conflict of interest.
