# Corrigendum: Dynamic Changes in DNA Methylation Occur during the First Year of Life in Preterm Infants

**DOI:** 10.3389/fendo.2018.00047

**Published:** 2018-02-27

**Authors:** Chinthika Piyasena, Jessy Cartier, Nadine Provencal, Tobias Wiechmann, Batbayar Khulan, Raju Sunderesan, Gopi Menon, Jonathan Seckl, Rebecca M. Reynolds, Elisabeth B. Binder, Amanda J. Drake

**Affiliations:** ^1^British Heart Foundation, Centre for Cardiovascular Science, The Queen’s Medical Research Institute, University of Edinburgh, Edinburgh, United Kingdom; ^2^Neonatal Unit, Simpson Centre for Reproductive Health, Royal Infirmary of Edinburgh, Edinburgh, United Kingdom; ^3^Department of Translational Research in Psychiatry, Max-Planck Institute of Psychiatry, Munich, Germany

**Keywords:** prematurity, DNA methylation, IGF2, FKBP5, glucocorticoids

An author name was incorrectly spelled as **[Sunderasan]**. The correct spelling is **[Sunderesan]**. The authors apologize for this error and state that this does not change the scientific conclusions of the article in any way.

The original article has been updated.

## Conflict of Interest Statement

The authors declare that the research was conducted in the absence of any commercial or financial relationships that could be construed as a potential conflict of interest.

